# Genomic and phenotypic characterisation of invasive neonatal and colonising group B Streptococcus isolates from Slovenia, 2001–2018

**DOI:** 10.1186/s12879-020-05599-y

**Published:** 2020-12-16

**Authors:** Tina Perme, Daniel Golparian, Maja Bombek Ihan, Andrej Rojnik, Miha Lučovnik, Lilijana Kornhauser Cerar, Petja Fister, Jana Lozar Krivec, Štefan Grosek, Alojz Ihan, Samo Jeverica, Magnus Unemo

**Affiliations:** 1 Department of Perinatology, University Medical Centre Ljubljana, Ljubljana, Slovenia; 2 WHO Collaborating Centre for Gonorrhoea and other STIs, National Reference Laboratory for STIs, Department of Laboratory Medicine, Microbiology, Örebro University, SE-70185 Örebro, Sweden; 3 National Laboratory for Health, Environment and Food, Maribor, Slovenia; 4 Department of Neonatology, Division of Pediatrics, University Medical Centre Ljubljana, Ljubljana, Slovenia; 5 Chair of Pediatrics, Faculty of Medicine, University of Ljubljana, Ljubljana, Slovenia; 6 Institute for Microbiology and Immunology, Medical Faculty, University of Ljubljana, Ljubljana, Slovenia

**Keywords:** Group B *Streptococcus*, GBS, Capsular type, Hypervirulent CC-17, Neonatal infection, Molecular epidemiology, Pathogenicity/virulence factors, Slovenia

## Abstract

**Background:**

Group B *Streptococcus* (GBS) is the leading cause of invasive neonatal disease in the industrialized world. We aimed to genomically and phenotypically characterise invasive GBS isolates in Slovenia from 2001 to 2018 and contemporary colonising GBS isolates from screening cultures in 2018.

**Methods:**

GBS isolates from 101 patients (invasive isolates) and 70 pregnant women (colonising isolates) were analysed. Basic clinical characteristics of the patients were collected from medical records. Antimicrobial susceptibility and phenotypic capsular serotype were determined. Whole-genome sequencing was performed to assign multilocus sequence types (STs), clonal complexes (CCs), pathogenicity/virulence factors, including capsular genotypes, and genome-based phylogeny.

**Results:**

Among invasive neonatal disease patients, 42.6% (*n* = 43) were females, 41.5% (*n* = 39/94) were from preterm deliveries (< 37 weeks gestation), and 41.6% (*n* = 42) had early-onset disease (EOD). All isolates were susceptible to benzylpenicillin with low minimum inhibitory concentrations (MICs; ≤0.125 mg/L). Overall, 7 serotypes were identified (Ia, Ib, II-V and VIII); serotype III being the most prevalent (59.6%). Twenty-eight MLST STs were detected that clustered into 6 CCs. CC-17 was the most common CC overall (53.2%), as well as among invasive (67.3%) and non-invasive (32.9%) isolates (*p* < 0.001). CC-17 was more common among patients with late-onset disease (LOD) (81.4%) compared to EOD (47.6%) (*p* < 0.001). The prevalence of other CCs was 12.9% (CC-23), 11.1% (CC-12), 10.5% (CC-1), 8.2% (CC-19), and 1.8% (CC-498). Of all isolates, 2.3% were singletons.

**Conclusions:**

A high prevalence of hypervirulent CC-17 isolates, with low genomic diversity and characteristic profile of pathogenicity/virulence factors, was detected among invasive neonatal and colonising GBS isolates from pregnant women in Slovenia. This is the first genomic characterisation of GBS isolates in Slovenia and provides valuable microbiological and genomic baseline data regarding the invasive and colonising GBS population nationally. Continuous genomic surveillance of GBS infections is crucial to analyse the impact of IND prevention strategies on the population structure of GBS locally, nationally, and internationally.

**Supplementary Information:**

The online version contains supplementary material available at 10.1186/s12879-020-05599-y.

## Background

Group B *Streptococcus* (GBS; *Streptococcus agalactiae*) is the leading cause of invasive neonatal disease (IND) in industrialized world [1]. IND is divided into early-onset disease (EOD), occurring within the first week postpartum, and late-onset disease (LOD), affecting infants aged > 1 week, mostly up to 90 days [2]. EOD can be prevented using intrapartum antibiotic prophylaxis. This is most effective when administered based on universal screening of GBS colonisation during the late third trimester of pregnancy or intrapartum [2]. In Slovenia, a less effective risk-based approach is predominantly used, which results in lower coverage of subsequent prophylaxis. This is likely the main reason for the high incidence of IND in Slovenia, estimated at 0.72/1000 live births, 0.53/1000 for EOD [3].

The polysaccharide capsule is the main pathogenicity/virulence factor of GBS [4]. Based on polysaccharide capsular antigens, GBS is divided into ten distinct serotypes (Ia, Ib, II-IX), which are antigenically and structurally distinct. The most common serotypes among GBS strains in Europe are serotypes Ia, II, III and V; with serotype III responsible for the majority of IND cases, particularly LOD [5, 6]. Additional pathogenicity/virulence factors have been implicated in the GBS colonisation and development of IND, among them several surface proteins such as pili, alpha-like proteins (ALP) family, C5a peptidase (ScpB), laminin-binding protein (Lmb), fibrinogen-binding proteins (Fbs), serine-rich proteins (Srr), and GBS immunogenic adhesins (Bib) [6, 7]. Pathogenicity/virulence factors of hypervirulent serotype III, multilocus sequence typing (MLST) clonal complex 17 (CC-17) isolates, have been particularly well studied and include serine-rich repeat glycoprotein 2 (Srr-2) and hypervirulent GBS adhesin (HvgA allele), conferring meningeal tropism contributing to the higher prevalence among LOD patients [8, 9].

For typing, whole genome sequencing (WGS) provides an ideal resolution and accuracy. However, simpler typing methods, such as MLST examining allelic variation in seven slowly evolving housekeeping genes, remain frequently used [10, 11]. Using MLST, bacterial isolates are classified into sequence types (ST), which cluster into CCs based on sequence similarities [10]. The majority of human GBS isolates cluster into 5 major CCs, namely CC-1, CC-12, CC-17, CC-19, and CC-23 [12]. An increase in the incidence of IND caused by the hypervirulent CC-17 has been previously described [12, 13]. The rapid expansion of CC-17 has been proposed to contribute to the limited success of current strategies to prevent IND in the industrialized world [13]. The WGS data additionally provide opportunities to characterise practically any other genotypic trait of bacterial isolates, such as the presence or absence of various pathogenicity/virulence factors, mutations, insertions, deletions or single nucleotide polymorphisms (SNPs).

In Slovenia, the prevalence of GBS colonisation among pregnant women is estimated at 17% [14], and very limited information is available about the epidemiology of neonatal GBS disease [3] and no data about the molecular epidemiology of GBS in the perinatal period exist. In the present study, all available Slovenian GBS isolates implicated in IND and a selection of contemporary colonising GBS isolates were phenotypically and genomically characterised.

## Methods

### Patients and bacterial isolates

This was a retrospective cohort study. Isolates from 101 neonates/infants (*n* = 114; invasive isolates) from 2001 to 2018 and 70 pregnant women (*n* = 71; colonising isolates) in 2018 were analysed. Invasive isolates were from blood (*n* = 96) and/or cerebrospinal fluid (CSF, *n* = 18) of neonates and infants aged 0–12 months. They were obtained from archived collections at all Slovenian microbiological laboratories (*n* = 4) (Supplementary Fig. 1). Based on the estimated incidence of IND in Slovenia [3], included cases represented 42% of all IND cases in Slovenia 2001–2018 (Supplementary Table 1). Basic demographic and clinical data were collected from the laboratory and hospital information systems. EOD was defined as occurring between 1 and 7 days postpartum, LOD between 8 and 90 days, and very late-onset disease (vLOD) between 91 and 365 days [2]. Colonising isolates were collected prospectively from consecutive vaginal (*n* = 52) or recto-vaginal (*n* = 19) screening swabs of pregnant women in 2018. All isolates were microbiologically characterized, however, only one isolate per patient was included in the analysis. If a patient had phenotypically identical GBS isolates cultured concomitantly from blood and CSF, the CSF isolate was included. Accordingly, blood isolates from 13 patients were excluded from the analysis, which resulted in the final number of 101 invasive GBS isolates. In the case of duplicate isolates from a woman in the colonisation group, only the first isolate was included in the analysis (one isolate was excluded, which resulted in the final number of 70 colonising isolates). Finally, invasive isolates were divided into 2 subgroups based on the year of isolation: the early isolates (isolated 2001–2011; isolates from the laboratory in Ljubljana lacking) and the late isolates (isolated 2012–2018) (Supplementary Fig. 1). This was mainly performed to examine changes in the Slovenian GBS population and especially if the number and proportion of serotype III and GBS CC-17 isolates increased over time. However, it was also performed because national coverage of GBS isolates was only available from 2012 and onwards. The study was approved by the National Medical Ethics Committee in Slovenia (KME 54/07/15).

### Phenotypic characterisation

Phenotypic characterisation was performed at the Institute of Microbiology and Immunology, Ljubljana, Slovenia. Species identification was performed by MALDI-TOF mass spectrometry (Bruker Daltonics, Bremen, Germany). Antibiotic susceptibility testing was performed and interpreted according to the EUCAST Clinical Breakpoint Tables v10.0 (www.eucast.org), using the disc diffusion method for vancomycin, levofloxacin, trimethoprim-sulfamethoxazole, erythromycin, clindamycin, and tetracycline on Mueller-Hinton fastidious agar. Minimum inhibitory concentrations (MICs) of benzylpenicillin and ampicillin were determined using the Etest (bioMérieux, Marcy l’Etoile, France) on Mueller-Hinton fastidious agar. Serotyping was conducted with ImmuLex Strep-B-Latex test (SSI Diagnostica, Hillerød, Danmark), as previously described [15]. After WGS-based ‘serotyping’ was available, all discrepant isolates were retested for the final result.

### Genomic characterisation

Genomic characterisation was performed at the WHO Collaborating Centre for Gonorrhoea and other STIs, Örebro University Hospital, Örebro, Sweden. Briefly, all isolates were grown from frozen stocks on blood agar media at 36 °C and bacterial suspensions were subjected to 60 min of lysis at 37 °C after adding an enzyme cocktail [16] containing lysozyme (20 mg/mL), mutanolysin (250 U/mL), and lysostaphin (20 U/mL) (Sigma-Aldrich, Saint Louis, Missouri, USA). Extraction of genomic DNA was performed using QIAsymphony DSP Virus/Pathogen Midi Kit (Qiagen, Hilden, Germany). Libraries were prepared using Nextera XT library preparation kit and WGS was performed on the Illumina MiSeq System (Illumina, San Diego, CA, USA) using Miseq Reagent kit V3 (600-cycle) producing 300 bp paired-end reads for each isolate with an average coverage of 126× per base (range: 82–180×). Reads were aligned to the chromosome of the *S. agalactiae* reference strain NEM316 (Genbank: NC_004368.1) using Burrows Wheeler Aligner (BWA) [17] with GATK indel realignment. Variant sites were identified from each isolate using bcftools (version 0.19) included in SAMtools (version 0.19) with default parameters [18] and filtered as described previously [19] to produce a multiple-sequence alignment.

De novo assembly was performed using CLC Genomics Workbench 12.0.1 and Velvet 1.2.10 assembler (https://github.com/dzerbino/velvet/tree/master) for confirmation [20]. MLST was performed from draft genomes and using the MLST tool (https://github.com/tseemann/mlst) as well as PubMLST (https://pubmlst.org). Clonal complexes were assigned using eBURST (http://eburst.mlst.net) [21]. Other genes of interest were extracted and characterised from the genome sequences using BLAST (https://blast.ncbi.nlm.nih.gov) and an in silico “PCR” method (https://github.com/egonozer/in_silico_pcr). WGS-based ‘serotyping’ was performed by analysing the variable region of the *cps* region [22].

Characterisations of surface and pathogenicity/virulence genes were performed in silico from draft genomes. Pili, ALP family (*alp1*, *rib*, *R28*, *alpha*), C5a peptidase (*scpB*), laminin/fibrinogen-binding proteins (*lmb, fbsA, fbsB*) and other adhesins (*bibA, hvgA*, *srr-1*, *srr-2*) genes were analysed. Previously described Pili, ALP, *srr* and *hvgA* genotypes [22] were determined using BLAST. For the genotypic characterisation of *scpB*, *lmb*, *fbsA* and *fbsB*, gene sequences were extracted from draft genomes, aligned with MUSCLE algorithm [23], and arbitrarily named using consecutive allele numbers. Neighbor-joining (NJ) trees were then constructed using SeaView 4.7 [24] and major clades were classified into allele numbers.

Phylogeny was achieved by mapping the reads to the reference genome of *S. agalactiae* NEM316 (NC_004368.1) using the bwa tool (http://bio-bwa.sourceforge.net) and constructing maximum-likelihood (ML) phylogenomic tree from the alignment using the generalized time reversible (GTR) substitution model and gamma distribution in the RAxML tool with 100 bootstraps (https://github.com/stamatak/standard-RAxML) [25]. Additionally, alignments were generated masked for recombination using the Gubbins tool (https://github.com/sanger-pathogens/gubbins [26]) and a second ML phylogenetic tree excluding regions of recombination was constructed. Phylogenetic trees were visualized with metadata using Microreact (https://microreact.org) and Phandango (https://jameshadfield.github.io/phandango/#/) [27, 28].

Raw sequence data were deposited at the European Nucleotide Archive (ENA); project accession number PRJEB35421.

### Statistical analysis

Descriptive statistics were used for sample characterisation. Chi-squared test was used for category proportion comparison between groups and subgroups. Significance was defined as *p*-values < 0.05.

## Results

### Patients and bacterial isolates

Basic patient characteristics are shown in Table 1. Briefly, 42.6% (*n* = 43) of patients were females, 41.5% (*n* = 39/94) were from preterm deliveries (< 37 weeks gestation), and 41.6% (*n* = 42) had EOD. Altogether, 171 patients/isolates were included in the analysis, 101 from neonates/infants with IND (invasive) and 70 from consecutive pregnant women (colonising).

**Table 1:** Basic patient information

Groups and categories	Number (%)
**PATIENTS**	
**Invasive disease**	**101 (100)**
***Gender***	
Female	43 (42.6)
Male	58 (57.4)
***Gestation*** (*n* = 94)	
Preterm (< 37 weeks)	39 (41.5)
Term (≥37 weeks)	55 (58.5)
***Disease type***^***a***^	
Early-onset (EOD)	42 (41.6)
Late-onset (LOD)	55 (54.5)
Very late-onset (vLOD)	4 (4)
***Geographical location***	
Ljubljana	42 (41.6)
Celje	25 (24.8)
Maribor	30 (29.7)
Koper	4 (4)
***Year of disease***	
2001–2011 (early)	31 (30.7)
2012–2018 (late)	70 (69.3)
**Colonisation**	**70 (100)**
***Age group***	
Age ≤ 30 years	35 (50)
Age > 30 years	35 (50)
***Geographical location***	
Ljubljana	70 (100)
***Year sample collection***	
2018	70 (100)
**Total**	**171 (100)**

^a^Disease type definition: EOD (1–7 days), LOD (8–90 days), vLOD (91–365 days)

### Antimicrobial susceptibility testing

All isolates were susceptible to benzylpenicillin, ampicillin, vancomycin, levofloxacin, and trimethoprim-sulfamethoxazole. The susceptibility to both erythromycin and clindamycin was > 80% (Supplementary Table 2). Most (87.2%, *n* = 149) isolates were resistant to tetracycline; invasive isolates (*n* = 93, 92.1%) resistant at higher frequency than colonising isolates (*n* = 56, 80%) (*p* = 0.02). None had elevated MICs (> 0.125 mg/L) of benzylpenicillin or ampicillin.

### Phenotypic and molecular ‘serotyping’

A pairwise comparison of conventional phenotypic serotyping and molecular ‘serotyping’ is summarised in Supplementary Table 3. A serotype could be phenotypically determined for all isolates (*n* = 171), while 4 isolates (2.3%) were non-typeable (NT) using the molecular method. Excluding the NT isolates, 87.4% (*n* = 146) of serotype results were concordant between the two methods. Nine, 5 and 7 isolates assigned the phenotypic serotypes Ia, Ib and III, respectively, gave discordant results in the molecular typing. Molecular serotype combined with phenotypic serotype for the 4 NT isolates was used as a final result. Overall, 7 capsular serotypes were identified (Ia, Ib, II, III, IV, V, and VIII). Serotype III was the most common serotype overall (59.6% of isolates), as well as among invasive isolates (74.3%) and colonising isolates (38.6%). However, the proportion of serotype III isolates was significantly higher among the invasive isolates compared to the colonising isolates (*p* < 0.001). The distribution of serotypes and CCs is depicted in Table 2.

**Table 2:** Distribution of serotypes and multilocus sequence typing MLST) clonal complexes (CC) among Slovenian group B *Streptococcus* isolates from patients with invasive neonatal infection and colonised pregnant woman in 2001–2018. One isolate per patient is included in the analysis (*n* = 171)

Serotype Clonal complex	All isolates	Invasive	Colonising
	*n* = 171	*n* = 101	*n* = 70
	No. (%)	No. (%)	No. (%)
**Ia**	**21 (12.3)**	**8 (7.9)**^**a**^	**13 (18.6)**^**a**^
CC-23	18 (10.5)	7 (6.9)	11 (15.7)
CC-1	1 (0.6)		1 (1.4)
CC-498	2 (1.2)	1 (1)	1 (1.4)
**Ib**	**12 (7)**	**7 (6.9)**	**5 (7.1)**
CC-12	12 (7)	7 (6.9)	5 (7.1)
**II**	**14 (8.2)**	**2 (2)**	**12 (17.1)**
CC-12	7 (4.1)	1 (1)	6 (8.6)
CC-19	5 (2.9)	1 (1)	4 (5.7)
CC-1	1 (0.6)		1 (1.4)
Singleton	1 (0.6)		1 (1.4)
**III**	**102 (59.6)**	**75 (74.3)**^**b**^	**27 (38.6)**^**b**^
CC-17	89 (52)	68 (67.3)	21 (30)
CC-19	8 (4.7)	6 (5.9)	2 (2.9)
CC-23	4 (2.3)	1 (1)	3 (4.3)
Singleton	1 (0.6)		1 (1.4)
**IV**	**2 (1.2)**		**2 (2.9)**
CC-17	2 (1.2)		2 (2.9)
**V**	**19 (11.1)**	**9 (8.9)**	**10 (14.3)**
CC-1	15 (8.8)	6 (5.9)	9 (12.9)
CC-19	1 (0.6)		1 (1.4)
CC-498	1 (0.6)	1 (1)	
Singleton	2 (1.2)	2 (2)	
**VIII**	**1 (0.6)**		**1 (1.4)**
CC-1	1 (0.6)		1 (1.4)
**Total**	**171 (100)**	**101 (100)**	**70 (100)**

*No.* number

^a^Proportions of serotype Ia isolates among invasive and colonising isolates were significantly different (*p* = 0.037)

^b^Proportions of serotype III isolates among invasive and colonising isolates were significantly different (*p* < 0.001)

### Multilocus sequence typing

Twenty-eight STs were detected, of which 10 had previously not been described. Thirteen and 24 unique STs were detected among the invasive and colonising isolates, respectively, showing higher variability within the latter (*p* < 0.001). Altogether, the STs were grouped into 6 CCs and 4 singletons based on eBURST analysis. CC-17, CC-23, CC-12, CC-1, and CC-19 included more than 10 isolates each. Overall, CC-17 was the most common CC, including 53.2% (*n* = 91) of isolates. CC-17 was more common among invasive versus colonising isolates (67.3 vs. 32.9%; *p* < 0.001), and LOD versus EOD isolates (81.4% vs. 47.6%; *p* < 0.001) (Supplementary Tables 4, 5 and 6). However, the proportion of CC-17 isolates was not significantly different (*p* = 0.187) in the early period (58.1%) compared to the late period (71.4%) (Supplementary Table 7).

### Phylogeny and characterisation of pathogenicity/virulence genes

**Fig. 1:**
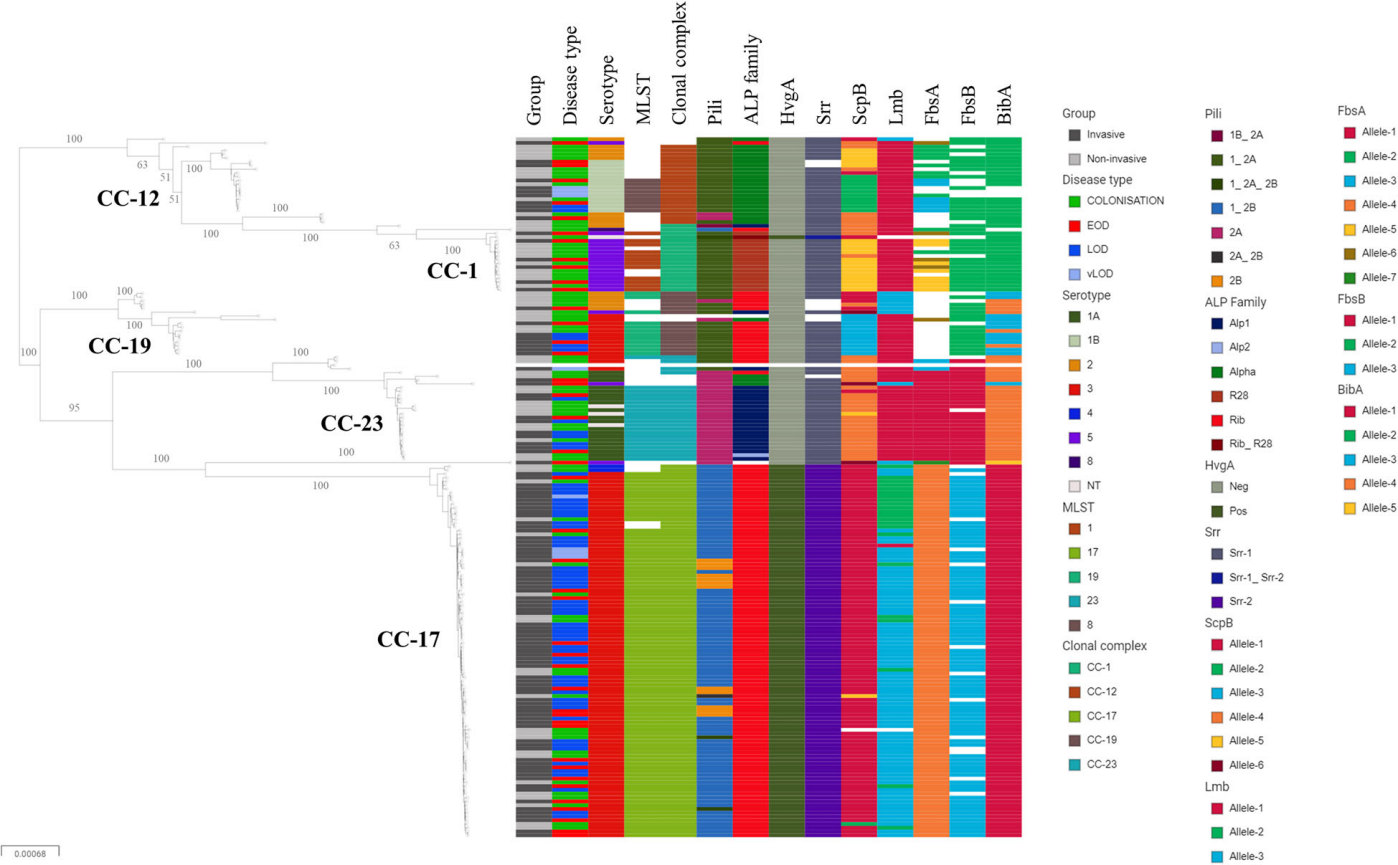
Single nucleotide polymorphism (SNP)-based maximum-likelihood phylogenomic tree with bootstrap values for the major branches including metadata: consisting of isolate group (invasive/colonising), disease type (early-onset/late-onset), serotype, MLST sequence type, MLST clonal complex, and surface/pathogenicity/virulence factors genotype (pili, alpha-like protein family, *hvgA*, *srr*, *scpB*, *lmb*, *fbsA*, *fbsB and bibA*). Colour of the bar depicts the genotype or lack of any named genotype or MLST sequence type or clonal complex (white bars). Pili, ALP, *srr* and *hvgA* genotypes were named in accordance with Metcalf et al. [22]. Alleles of *scpB*, *lmb*, *fbsA* and *fbsB* were arbitrarily assigned consecutive numbers

A SNP-based ML phylogenetic tree including metadata is shown in Fig. 1. Six clades with ≥5 isolates could be distinguished within the 5 major CCs. CC-19 was represented by 2 clades characterised by different serotypes, i.e. II and III. The majority (4/5, 80%) of these serotype II isolates were colonising and the serotype III isolates were predominantly invasive (*n* = 7/9, 78%). Overall, CC-17 isolates were almost exclusively assigned serotype III and they were predominantly invasive. However, two colonising CC-17 isolates were of serotype IV. A high homogeneity of surface and pathogenicity/virulence factors was observed within the CCs. As almost one third (32.9%) of non-invasive colonising isolates belonged to CC-17, it was difficult to compare the presence/absence of different pathogenicity/virulence factors between the invasive and colonising isolates. Typical profiles of pathogenicity/virulence factors of the 5 most common CCs are depicted in Table 3.

**Table 3:** Pathogenicity/virulence factors in group B *Streptococcus* isolates, belonging to the 5 major multilocus sequence typing (MLST) clonal complexes (CCs), cultured in Slovenia from 2001 to 2018. The most prevalent genotype within each CC and its proportion are shown

Clonal complex	Number (%)	Capsule (*cps*) [22]	Pili [22]	ALP family (*alp1*, *alpha*, *rib*, *R28*) [22]	C5a peptidase (*scpB*)	Laminin-binding protein (*lmb*)	Fibrinogen-binding protein A (*fbsA*)	Fibrinogen-binding protein B (*fbsB*)	Serine-rich proteins (*srr*) [22]	GBS immunogenic adhesin A (*bibA*)	Hypervirulent GBS adhesin (*hvgA* allele)
		Genotype (%)									
**CC-1**	18 (10.5)	V (83.3)	1, 2A (83.3)	R28 (83.3)	allele-5 (72.2)	allele-1 (94.4)	allele-5 (44.4)	allele-2 (88.9)	srr-1 (94.4)	allele-2 (94.4)	neg (94.4)
**CC-12**	19 (11.1)	Ib (63.2)	1, 2A (89.5)	alpha (100)	allele-2 (42.1)	allele-1 (100)	allele-2 (36.8)	allele-2 (68.4)	srr-1 (89.5)	allele-2 (94.7)	neg (100)
**CC-17**	91 (53.2)	III (97.8)	1, 2B (84.6)	rib (100)	allele-1 (96.7)	allele-3 (74.7)	allele-4 (100)	allele-3 (85.7)	srr-2 (100)	allele-1 (100)	pos (100)
**CC-19**	14 (8.2)	III (57.1)	1, 2A (92.9)	rib (92.9)	allele-3 (57.1)	allele-1 (57.1)	null (100)	allele-2 (78.6)	srr-1 (78.6)	allele-3 (57.1)	neg (100)
**CC-23**	22 (12.9)	Ia (81.8)	2A (86.4)	alp1 (81.8)	allele-4 (90.9)	allele-1 (100)	allele-1 (86.4)	allele-1 (90.9)	srr-1 (100)	allele-4 (100)	neg (100)

*ALP* alpha-like proteins, *neg* negative, *pos* positive

Pili, ALP, *srr* and *hvgA* genotypes were named in accordance with Metcalf et al. [22]. Alleles of *scpB*, *lmb*, *fbsA* and *fbsB* were arbitrarily assigned consecutive numbers

**Fig. 2:**
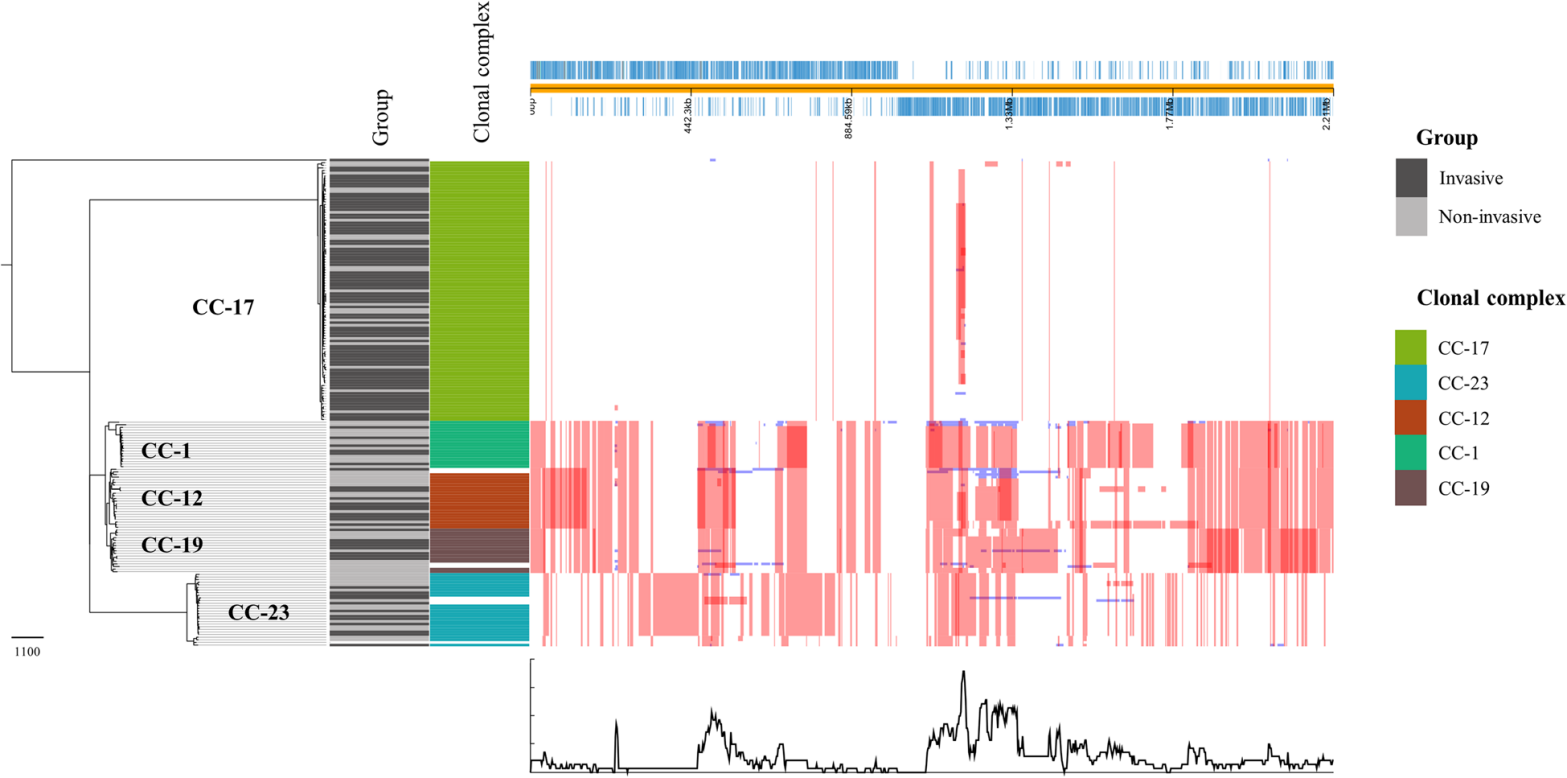
Single nucleotide polymorphism (SNP)-based maximum-likelihood phylogenomic tree after regions of recombination have been excluded using Gubbins [26]. Group (invasive/non-invasive) and MLST clonal complex are described for each isolate and white bars depict isolates that do not belong to any of the five named major MLST clonal complexes. Genomic regions with high frequency of recombination are mapped to the reference genome of *Streptococcus agalactiae* NEM316 (annotated in blue on top). Each row represents an isolate and the columns relate to bases in the reference genome. The red columns are recombinations shared by multiple isolates and occuring in the internal branches. The blue columns are recombinations in the terminal branch and represented by unique isolates

SNP-based ML phylogenetic tree was also constructed after excluding regions of abundant recombination using Gubbins [26] (Fig. 2).

A striking difference in frequency of recombination within CC-17 isolates compared to isolates of other CCs was observed (Fig. 2). This highlights the importance of horizontal gene transfer and recombination in GBS, especially among non-CC-17 strains. In contrast, the CC-17 hypervirulent clade had few regions prone to recombination.

## Discussion

In this first molecular epidemiology and genomic study of GBS in Slovenia, we show a high prevalence of hypervirulent MLST CC-17 among invasive isolates (67.3%), but also among contemporary colonising (32.9%) isolates. The CC-17 isolates were relatively conserved genomically and mostly belonged to serotype III. Slovenian GBS isolates were uniformly susceptible to benzylpenicillin (MICs≤0.125 mg/L), whereas the resistance to erythromycin (17%) and clindamycin (16%) was comparable to that of other European countries [29, 30].

The concordance between phenotypic and molecular ‘serotyping’ methods was 87%, suggesting imperfect but mainly sufficient typing using also sequencing methods, particularly in view of the increasing availability of WGS and other molecular methods [22]. This concordance is also in line with two recent studies, that is, describing 87–94% concordance [31, 32]. Nevertheless, this suboptimal concordance is important to take into account when performing, for example, surveillance studies informing vaccine design. Overall, 7 serotypes were identified, with serotype III accounting for the majority of isolates (60%). Serotype III isolates mostly belonged to CC-17 (52%), but some were assigned CC-19 (4.7%) and CC-23 (2.3%). Serotype III was predominantly associated with invasive disease (74% of invasive isolates). Serotypes among colonising isolates were more evenly distributed, consistent with data from a recent meta-analysis [33].

GBS isolates in our study displayed a high level of genomic diversity with 28 MLST STs detected, 9 of which had not been described previously. The diversity was larger among the colonising isolates. Nevertheless, CC-17 comprised more than half of all isolates and was more common among the invasive and LOD isolates. This hypervirulent clone also showed a trend towards higher prevalence among the late subgroup of isolates (2012–2018) (58% vs. 71%), similar to a study from the Netherlands [13]. CC-17 had a characteristic profile of pathogenicity/virulence factors that included serotype III, pili 1-2B, ALP family *rib*, *scpB* allele-1, *fbsA* allele-4, *fbsB* allele-3, *srr-2*, *bibA* allele-1 and *hvgA* positive. These results are in-line with several previous studies [7–9, 13, 27].

The genome organisation of the frequently invasive CC-17 isolates was highly conserved with few recombination prone regions. This may indicate that CC-17 has already experienced an evolutionary selection to increase fitness for survival and pathogenicity/virulence. In contrast, non-CC-17 isolates were recombination prone, highlighting the importance of recombination and horizontal gene transfer in GBS evolution [12]. Interestingly, CC-1, CC-12 and CC-19, which are predominantly colonising CCs, belonged to the same clade after the regions of recombination were removed (Fig. 2).

The limitations of the present study included that we were not able to include isolates from all cases of IND due to the unavailability of GBS isolates from 2001 to 2010 in the largest Slovenian laboratory (in Ljubljana). Furthermore, colonising isolates were available only from 2018 and the laboratory in Ljubljana. Finally, we had limited clinical data from the IND cases. However, despite these limitations, a relatively large number of IND cases, isolates and standard genomic analysis tools provided us with detailed and reliable baseline information about the GBS population structure in Slovenia.

## Conclusions

A high prevalence of hypervirulent CC-17 isolates, with low genomic diversities and characteristic profile of pathogenicity/virulence factors, was detected among invasive neonatal and colonising GBS isolates from pregnant women in Slovenia. This is the first genomic characterisation of GBS isolates in Slovenia and provides valuable microbiological and genomic baseline data regarding the invasive and colonising GBS population in Slovenia. Continuous genomic surveillance of GBS infections is crucial to analyse the impact of IND prevention strategies on the population structure of GBS locally, nationally and internationally.

## Supplementary Information

Additional file 1: **Supplementary Table 1.** Number of births in Slovenia during the years 2002–2018 and calculated representativeness of the sample based on the estimated incidence of invasive neonatal disease (IND) of 0.7/1000 births from reference 3. **Supplementary Table 2.** Antimicrobial susceptibility of Slovenian invasive neonatal and colonising pregnant women isolates of group B *Streptococcus* from early (2001–2011) and late (2012–2018) period (*n* = 171)*.* **Supplementary Table 3.** Pairwise comparison of conventional phenotypic serotyping and molecular ‘serotyping’ results among invasive and non-invasive isolates of group B *Streptococcus* from early (2001–2011) and late (2012–2018) period (*n =* 171)*.* **Supplementary Table 4.** Distribution of multilocus sequence typing (MLST) sequence types (STs) and clonal complexes (CCs) among Slovenian group B *Streptococcus* isolates overall and among isolates from infants with invasive neonatal disease (2001–2018) and pregnant woman with colonisation (2018. **Supplementary Table 5.** Distribution of multilocus sequence typing (MLST) and clonal complexes (CCs) among Slovenian group B *Streptococcus* isolates overall and among isolates from patients with invasive neonatal disease (2001–2018) and pregnant woman with colonisation (2018). **Supplementary Table 6.** Distribution of multilocus sequence typing (MLST) and clonal complexes (CCs) among invasive neonatal group B *Streptococcus* isolates from Slovenia within two groups based on disease onset.** Supplementary Table 7.** Distribution of multilocus sequence typing (MLST) and clonal complexes (CCs) among invasive neonatal group B *Streptococcus* isolates from Slovenia within two groups based on time period of disease. **Supplementary Fig. 1*.*** Distribution of invasive group B *Streptococcus* isolates (*n* = 114) from different laboratories in Slovenia from 2001 to 2018. Unfortunately, during 2001–2010, isolates from the largest laboratory (in Ljubljana) were not preserved so whole genome sequencing of those could not be performed. LJ: Ljubljana; MB: Maribor; CE: Celje; KP: Koper. **Supplementary Fig. 2*.*** eBURST analysis of 28 multilocus sequence typing (MLST) sequence types (STs) identifying six clonal complexes (CCs) and four singletons, among Slovenian group B *Streptococcus* isolates from infants with invasive neonatal disease (2001–2018) and pregnant woman with colonisation (2018).

## Data Availability

The datasets used and/or analysed during the current study are available from the corresponding author on reasonable request.

## Abbreviations

ALP: Alpha like proteins
Bib: GBS immunogenic adhesins
CC: Clonal complex
CSF: Cerebrospinal fluid
EOD: Early onset disease
Fbs: Fibrinogen binding proteins
GBS: Group B *Streptococcus*
HvgA: Hypervirulent GBS adhesin
IND: Invasive neonatal disease
Lmb: Laminin binding protein
LOD: Late onset disease
MALDI-TOF: Matrix assisted laser desorption/ionization-time of flight
MIC: Minimum inhibitory concentration
MLST: Multilocus sequence typing
NT: Non-typeable
ScpB: C5a peptidase
SNP: Single nucleotide polymorphism
Srr: Serine rich protein
ST: Sequence type
vLOD: Very late onset disease
WGS: Whole genome sequencing
