# Supplementary material for: Genomic and phenotypic characterisation of invasive neonatal and colonising group B Streptococcus isolates from Slovenia, 2001–2018
Source: BMC Infect Dis. 2020 Dec 16;20:958. doi: 10.1186/s12879-020-05599-y (PMC7739447; doi:10.1186/s12879-020-05599-y)
Supplement: Supplementary file 1 — Supplementary Table 1. Number of births in Slovenia during the years 2002–2018 and calculated representativeness of the sample based on the estimated incidence of invasive neonatal disease (IND) of 0.7/1000 births from reference 3. Supplementary Table 2. Antimicrobial susceptibility of Slovenian invasive neonatal and colonising pregnant women isolates of group B Streptococcus from early (2001–2011) and late (2012–2018) period (n = 171). Supplementary Table 3. Pairwise comparison of conventional phenotypic serotyping and molecular ‘serotyping’ results among invasive and non-invasive isolates of group B Streptococcus from early (2001–2011) and late (2012–2018) period (n = 171). Supplementary Table 4. Distribution of multilocus sequence typing (MLST) sequence types (STs) and clonal complexes (CCs) among Slovenian group B Streptococcus isolates overall and among isolates from infants with invasive neonatal disease (2001–2018) and pregnant woman with colonisation (2018. Supplementary Table 5. Distribution of multilocus sequence typing (MLST) and clonal complexes (CCs) among Slovenian group B Streptococcus isolates overall and among isolates from patients with invasive neonatal disease (2001–2018) and pregnant woman with colonisation (2018). Supplementary Table 6. Distribution of multilocus sequence typing (MLST) and clonal complexes (CCs) among invasive neonatal group B Streptococcus isolates from Slovenia within two groups based on disease onset. Supplementary Table 7. Distribution of multilocus sequence typing (MLST) and clonal complexes (CCs) among invasive neonatal group B Streptococcus isolates from Slovenia within two groups based on time period of disease. Supplementary Fig. 1. Distribution of invasive group B Streptococcus isolates (n = 114) from different laboratories in Slovenia from 2001 to 2018. Unfortunately, during 2001–2010, isolates from the largest laboratory (in Ljubljana) were not preserved so whole genome sequencing of those could not be performed. LJ [file 12879_2020_5599_MOESM1_ESM.docx]

**Supplementary file**: **Supplementary tables 1-6.** **Supplementary figures 1 and 2.**

**Supplementary table 1.** Number of births in Slovenia during the years 2002-2018 and calculated representativeness of the sample based on the estimated incidence of invasive neonatal disease (IND) of 0.7/1000 births from reference 3

| **YEAR** | **BIRTHS (No.)** |
| --- | --- |
| 2002 | 17,583 |
| 2003 | 17,160 |
| 2004 | 17,900 |
| 2005 | 18,199 |
| 2006 | 19,029 |
| 2007 | 19,915 |
| 2008 | 21,866 |
| 2009 | 21,763 |
| 2010 | 22,296 |
| 2011 | 21,846 |
| 2012 | 21,789 |
| 2013 | 20,875 |
| 2014 | 20,857 |
| 2015 | 20,280 |
| 2016 | 20,083 |
| 2017 | 19,947 |
| 2018 | 19,329 |
| **TOTAL** | **34,0717** |
| ALL ISOLATES (estimated) = **POPULATION** | **238** |
| AVAILABLE ISOLATES (study) = **SAMPLE** | **101** |
| PROPORTION = **REPRESENTATIVENESS** | **42**% |

No., number

**Supplementary table 2.** Antimicrobial susceptibility of Slovenian invasive neonatal and colonising pregnant women isolates of group B Streptococcus from early (2001-2011) and late (2012-2018) period (n=171)

| **Antibiotic** | **Susceptible, % (Number)** |  |  |  |  |
| --- | --- | --- | --- | --- | --- |
|  | **Total** | **Invasive** |  |  | **Colonising** |
| **Year** |  | All | Early period | Late period | 2018 |
|  | n=171 | n=101 | n=31 | n=70 | n=70 |
| Benzylpenicillin | 100 (171) | 100 (101) | 100 (31) | 100 (70) | 100 (70) |
| Ampicillin | 100 (171) | 100 (101) | 100 (31) | 100 (70) | 100 (70) |
| Vancomycin | 100 (171) | 100 (101) | 100 (31) | 100 (70) | 100 (70) |
| Levofloxacin | 100 (171) | 100 (101) | 100 (31) | 100 (70) | 100 (70) |
| TMP-SMX | 100 (171) | 100 (101) | 100 (31) | 100 (70) | 100 (70) |
| Erythromycin | 82.5 (141) | 83.1 (84) | 90.3 (28) | 80 (56) | 81.4 (57) |
| Clindamycin | 83.6 (143) | 81.2 (82) | 87.1 (27) | 78.6 (55) | 87.1 (61) |
| Tetracycline | 12.9 (22) | 7.9 (8) | 3.2 (1) | 10 (7) | 20 (14) |

TMP-SMX: trimethoprim-sulfamethoxazole

**Supplementary table 3.** Pairwise comparison of conventional phenotypic serotyping and molecular ‘serotyping’ results among invasive and non-invasive isolates of group B Streptococcus from early (2001-2011) and late (2012-2018) period (n=171)


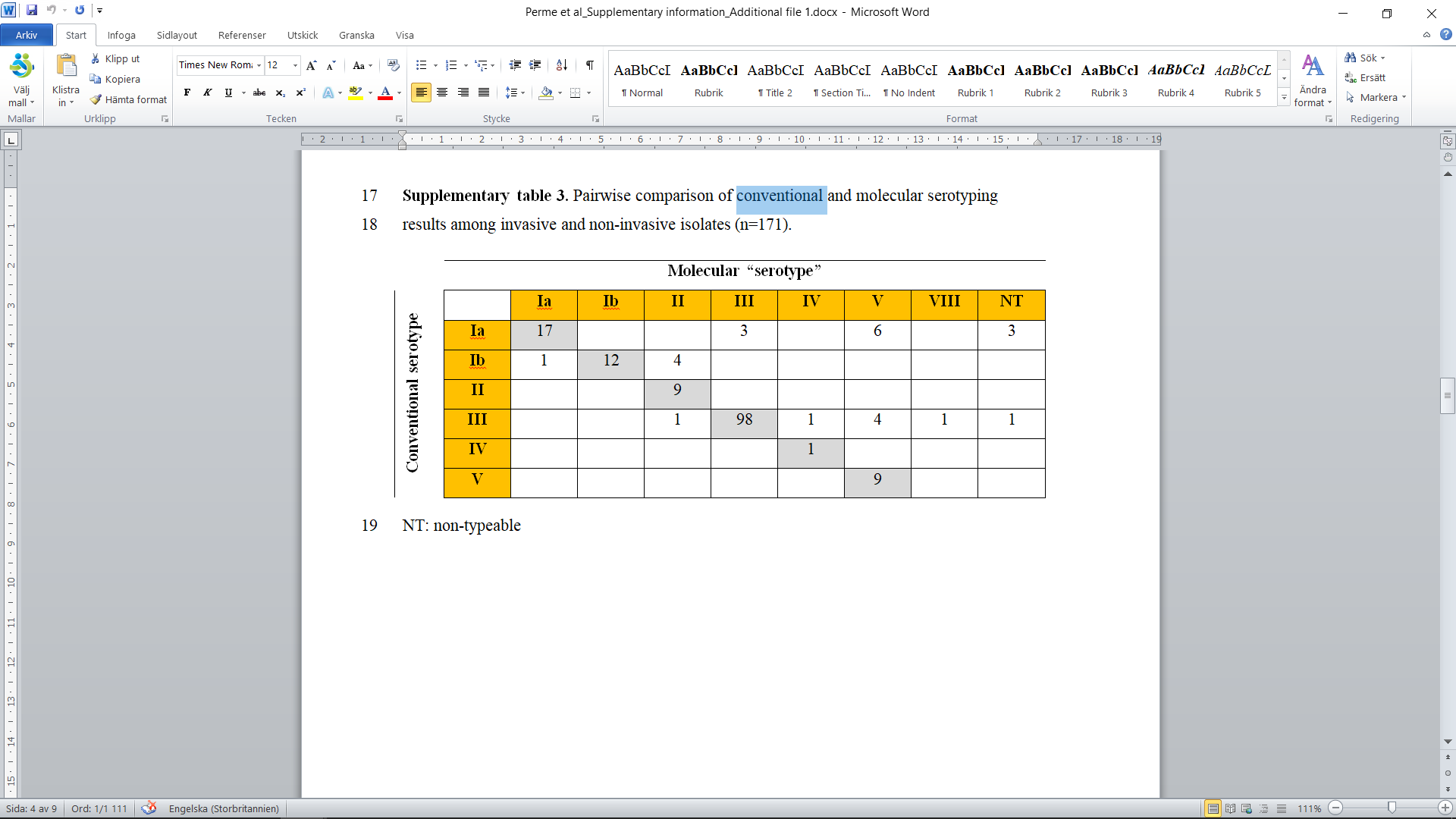


NT, non-typeable

**Supplementary table 4.** Distribution of multilocus sequence typing (MLST) sequence types (STs) and clonal complexes (CCs) among Slovenian group B Streptococcus isolates overall and among isolates from infants with invasive neonatal disease (2001-2018) and pregnant woman with colonisation (2018)

| **Clonal** **complex / sequence type** | **All isolates**  (n=171) | **Invasive isolates**  (n=101) | **Colonising isolates**  (n=70) |
| --- | --- | --- | --- |
|  | No. (%) | No. (%) | No. (%) |
| **CC-1** | **18 (10.5)** | **6 (5.9)** | **12 (17.1)** |
| ST-1 | 12 (7) | 6 (5.9) | 6 (8.6) |
| ST-2 | 1 (0.6) |  | 1 (1.4) |
| ST-1431^a^ | 1 (0.6) |  | 1 (1.4) |
| ST-1426^a^ | 1 (0.6) |  | 1 (1.4) |
| ST-1430^a^ | 1 (0.6) |  | 1 (1.4) |
| ST-1425^a^ | 2 (1.2) |  | 2 (2.9) |
| **CC-12** | **19 (11.1)** | **8 (7.9)** | **11 (15.7)** |
| ST-12 | 7 (4.1) | 3 (3) | 4 (5.7) |
| ST-8 | 7 (4.1) | 5 (5) | 2 (2.9) |
| ST-9 | 2 (1.2) |  | 2 (2.9) |
| ST-1428^a^ | 1 (0.6) |  | 1 (1.4) |
| ST-1424^a^ | 1 (0.6) |  | 1 (1.4) |
| ST-1427^a^ | 1 (0.6) |  | 1 (1.4) |
| **CC-17** | **91 (53.2)** | **68 (67.3)** | **23 (32.9)** |
| ST-17 | 87 (50.9) | 66 (65.3) | 21 (30) |
| ST-1423^a^ | 2 (1.2) | 2 (2) |  |
| ST-291 | 2 (1.2) |  | 2 (2.9) |
| **CC-19** | **14 (8.2)** | **7 (6.9)** | **7 (10)** |
| ST-19 | 11 (6.4) | 6 (5.9) | 5 (7.1) |
| ST-28 | 2 (1.2) | 1 (1) | 1 (1.4) |
| ST-267 | 1 (0.6) |  | 1 (1.4) |
| **CC-23** | **22 (12.9)** | **8 (7.9)** | **14 (20)** |
| ST-23 | 19 (11.1) | 7 (6.9) | 12 (17.1) |
| ST-477 | 1 (0.6) | 1 (1) |  |
| ST-144 | 1 (0.6) |  | 1 (1.4) |
| ST-1429^a^ | 1 (0.6) |  | 1 (1.4) |
| **CC-498** | **3 (1.8)** | **2 (2)** | **1 (1.4)** |
| ST-498 | 2 (1.2) | 1 (1) | 1 (1.4) |
| ST-24 | 1 (0.6) | 1 (1) |  |
| **Singleton** | **4 (2.3)** | **2 (2)** | **2 (2.9)** |
| ST-26 | 1 (0.6) | 1 (1) |  |
| ST-41 | 1 | 1 (1) |  |
| ST-529 | 1 |  | 1 (1.4) |
| ST-569 | 1 |  | 1 (1.4) |
| **Grand Total** | **171 (100)** | **101 (100)** | **70 (100)** |

No., number;
^a^Novel sequence types

**Supplementary table 5.** Distribution of multilocus sequence typing (MLST) and clonal complexes (CCs) among Slovenian group B Streptococcus isolates overall and among isolates from patients with invasive neonatal disease (2001-2018) and pregnant woman with colonisation (2018)

| **Group** / **clonal** **complex** | **All isolates** |  |
| --- | --- | --- |
|  | **Relative** **proportions** (within group) | **Absolute** **proportion** (within all isolates) |
|  | n=171 | n=171 |
|  | No. (%) | No. (%) |
| **Overall** | **/** | **171 (100)** |
| CC-17 | / | 91 (53.2) |
| CC-23 | / | 22 (12.9) |
| CC-12 | / | 19 (11.1) |
| CC-1 | / | 18 (10.5) |
| CC-19 | / | 14 (8.2) |
| singleton | / | 4 (2.3) |
| CC-498 | / | 3 (1.8) |
| **Invasive** | **101 (100)** | **101 (59.1)** |
| CC-17 | 68 (67.3)^a^ | 68 (39.8) |
| CC-23 | 8 (7.9) | 8 (4.7) |
| CC-12 | 8 (7.9) | 8 (4.7) |
| CC-19 | 7 (6.9) | 7 (4.1) |
| CC-1 | 6 (5.9) | 6 (3.5) |
| singleton | 2 (2) | 2 (1.2) |
| CC-498 | 2 (2) | 2 (1.2) |
| **Colonising** | **70 (100)** | **70 (40.9)** |
| CC-17 | 23 (32.9)^a^ | 23 (13.5) |
| CC-23 | 14 (20) | 14 (8.2) |
| CC-1 | 12 (17.1) | 12 (7) |
| CC-12 | 11 (15.7) | 11 (6.4) |
| CC-19 | 7 (10) | 7 (4.1) |
| singleton | 2 (2.9) | 2 (1.2) |
| CC-498 | 1 (1.4) | 1 (0.6) |
| **Total** | **171 (100)** | **171 (100)** |

No., number

^a^The difference between proportions of CC-17 isolates among invasive and colonising group was statistically significant (*p*<0.001).

**Supplementary table 6.** Distribution of multilocus sequence typing (MLST) and clonal complexes (CCs) among invasive neonatal group B Streptococcus isolates from Slovenia within two groups based on disease onset

| **Group** / **clonal** **complex** | **All isolates** |  |
| --- | --- | --- |
|  | **Relative** **proportions** (within group) | **Absolute** **proportion** (within all isolates) |
|  | n=101 | n=101 |
|  | No. (%) | No. (%) |
| **Early-onset disease (EOD)** | **42 (100)** | **42 (40.6)** |
| CC-17 | 20 (47.6)^a^ | 20 (19.8) |
| CC-1 | 6 (14.3) | 6 (5.9) |
| CC-12 | 5 (11.9) | 5 (5) |
| CC-19 | 4 (9.5) | 4 (4) |
| CC-23 | 3 (7.1) | 3 (3) |
| singleton | 2 (4.8) | 2 (2) |
| CC-498 | 2 (4.8) | 2 (2) |
| **Late-onset disease (LOD)** | **59 (100)** | **59 (58.4)** |
| CC-17 | 48 (81.4)^a^ | 48 (47.5) |
| CC-23 | 5 (8.5) | 5 (5) |
| CC-12 | 3 (5.1) | 3 (3) |
| CC-19 | 3 (5.1) | 3 (3) |
| **Total** | **101 (100)** | **101 (100)** |
|  |  |  |

No., number

*The difference between proportions of CC-17 isolates among early- and late-onset disease was statistically significant (*p*<0.001).

**Supplementary table 7.** Distribution of multilocus sequence typing (MLST) and clonal complexes (CCs) among invasive neonatal group B Streptococcus isolates from Slovenia within two groups based on time period of disease

| **Group** / **clonal** **complex** | **All isolates** |  |
| --- | --- | --- |
|  | **Relative** **proportions** (within group) | **Absolute** **proportion** (within all isolates) |
|  | n=101 | n=101 |
|  | No. (%) | No. (%) |
| **≤2011 (early period)** | **31 (100)** | **31 (30.7)** |
| CC-17 | 18 (58.1) | 18 (17.8) |
| CC-19 | 5 (16.1) | 5 (5) |
| CC-12 | 4 (12.9) | 4 (4) |
| CC-1 | 2 (6.5) | 2 (2) |
| CC-498 | 1 (3.2) | 1 (1) |
| CC-23 | 1 (3.2) | 1 (1) |
| **≥2012 (late period)** | **70 (100)** | **70 (69.3)** |
| CC-17 | 50 (71.4) | 50 (49.5) |
| CC-23 | 7 (10) | 7 (6.9) |
| CC-1 | 4 (5.7) | 4 (4) |
| CC-12 | 4 (5.7) | 4 (4) |
| singleton | 2 (2.9) | 2 (2) |
| CC-19 | 2 (2.9) | 2 (2) |
| CC-498 | 1 (1.4) | 1 (1) |
| **Total** | **101 (100)** | **101 (100)** |

**
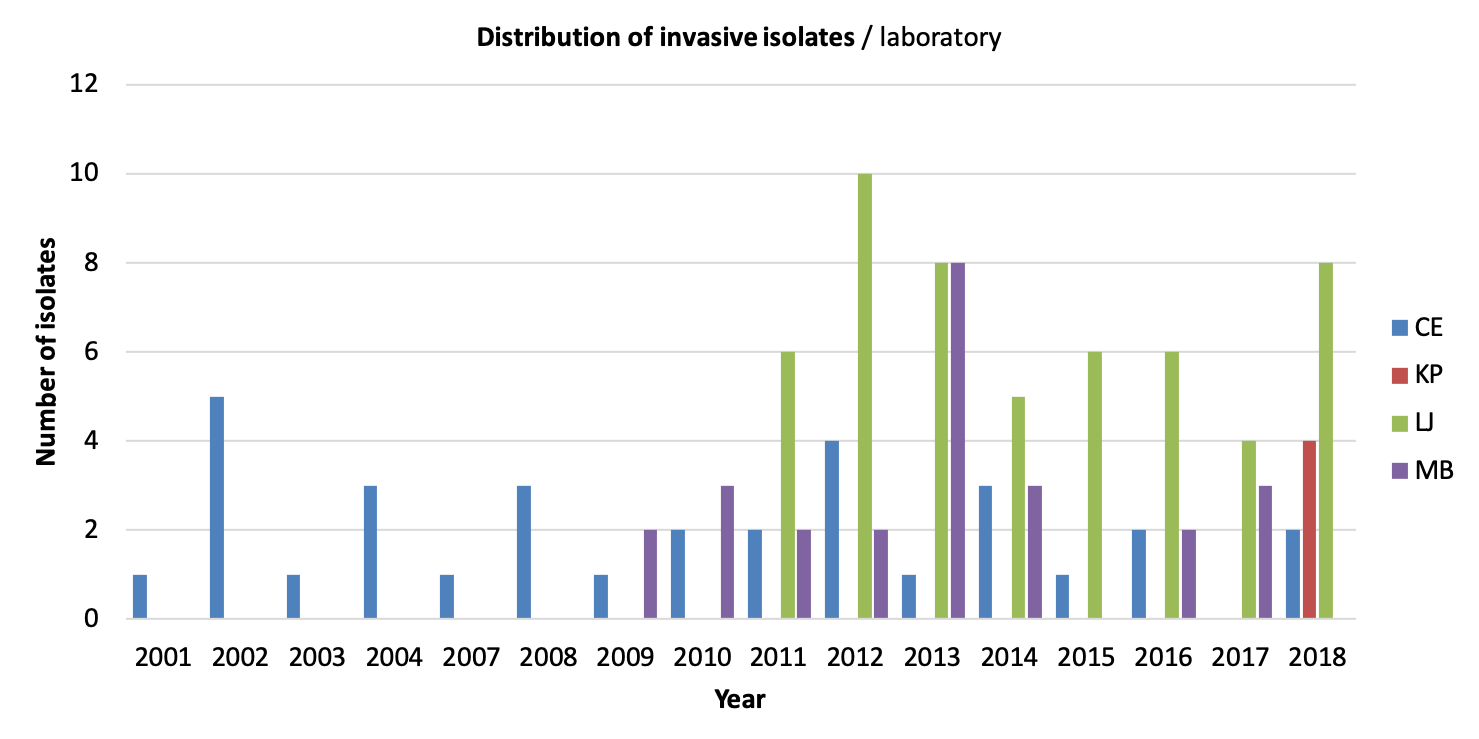
**

**Supplementary figure 1*.*** Distribution of invasive group B *Streptococcus* isolates (n=114) from different laboratories in Slovenia from 2001 to 2018. Unfortunately, during 2001-2010, isolates from the largest laboratory (in Ljubljana) were not preserved so whole genome sequencing of those could not be performed. LJ: Ljubljana; MB: Maribor; CE: Celje; KP: Koper


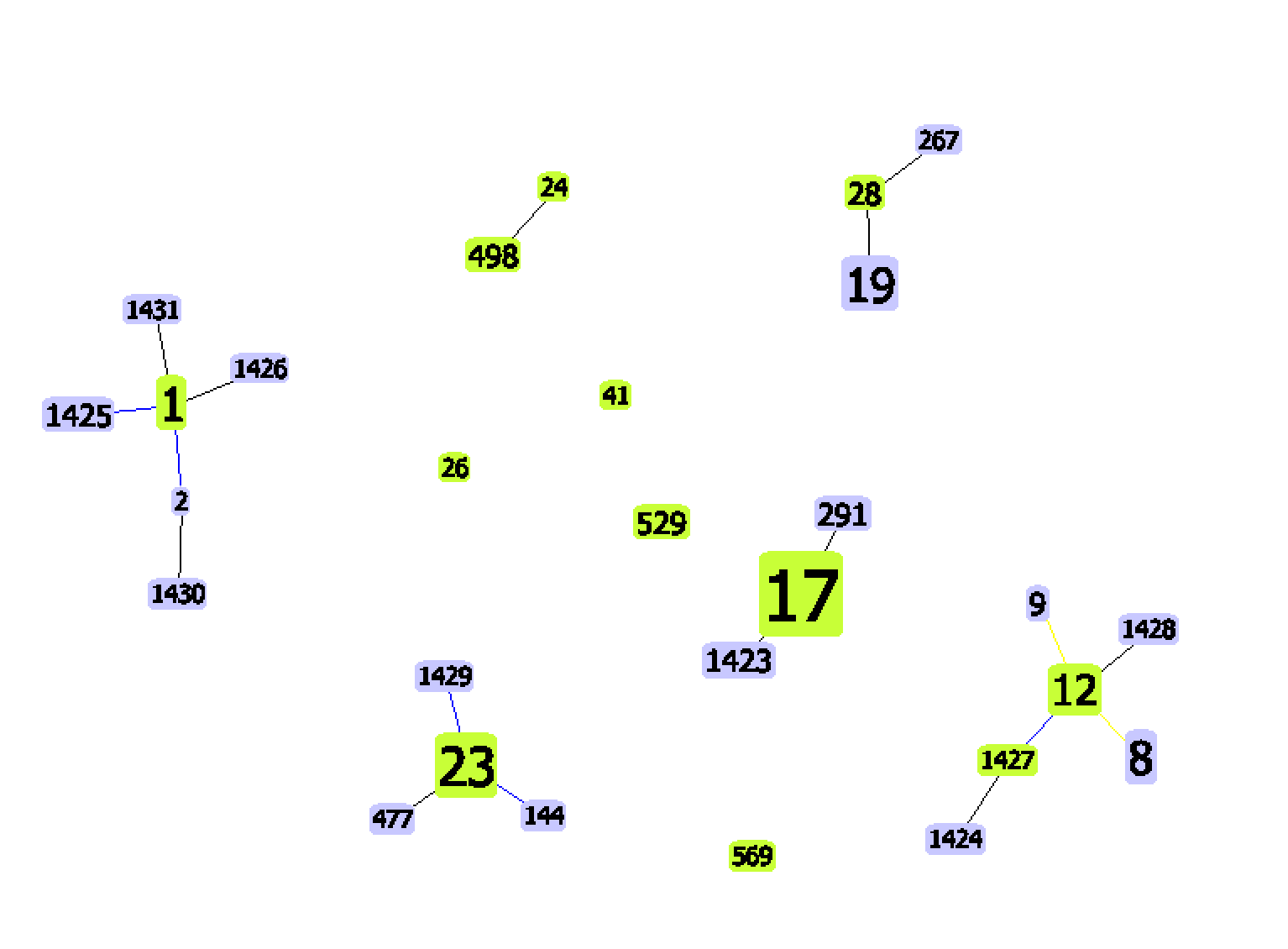


**Supplementary figure 2*.*** eBURST analysis of 28 multilocus sequence typing (MLST) sequence types (STs) identifying six clonal complexes (CCs) and four singletons, among Slovenian group B Streptococcus isolates from infants with invasive neonatal disease (2001-2018) and pregnant woman with colonisation (2018).
